# Clinical significance of measuring plasminogen activator inhibitor-1 in sepsis

**DOI:** 10.1186/s40560-017-0250-z

**Published:** 2017-09-06

**Authors:** Toshiaki Iba, Jecko Thachil

**Affiliations:** 1 Department of Emergency and Disaster Medicine, Juntendo University Graduate School of Medicine, 2-1-1 Hongo Bunkyo-ku, Tokyo, 113-8421 Japan; 2 Department of Haematology, Manchester Royal Infirmary, Manchester, UK

**Keywords:** Sepsis, Plasminogen activator inhibitor-1, Disseminated intravascular coagulation

## Abstract

**Background:**

Disseminated thrombotic process in the microcirculation is considered to be an important cause of multiple organ dysfunction in sepsis. The fundamental purpose of this prothrombotic change was believed to be in the host defense against microbial dissemination. In that process, antifibrinolytic property plays an important role.

**Main body:**

For the understanding of pathophysiology of sepsis, it is quite useful to grasp the alterations in coagulation/fibrinolytic parameters, i.e., plasminogen activator and plasminogen activator inhibitor-1. They play crucial roles in the development of clot formation and disseminated intravascular coagulation that leads to fatal organ dysfunction. Basically, fibrinolysis is a simple system compared to the complex coagulation cascade. Plasmin is the only factor that regulates fibrinolysis, and this enzyme is modulated by several factors including plasminogen activators and plasminogen activator inhibitor-1. However, recent studies have elucidated the complex regulation of the production, activation, and inactivation of these fibrinolytic factors.

**Conclusion:**

The dynamic change of the fibrinolytic system plays a crucial role in the pathophysiology of sepsis. In this commentary, we introduce the recent advances of the research regarding fibrinolytic system.

## Background

It is well known that fibrin removal is severely hindered by inactivation of the endogenous fibrinolytic system, mainly as a result of upregulation of plasminogen activator inhibitor type-1 (PAI-1). Consequent increased fibrin generation and impaired fibrin breakdown leads to deposition of clots in the microvasculature, leading to the tissue ischemia and ensuing organ dysfunction. Numerous experimental and clinical studies have revealed that the levels of PAI-1 but not tissue plasminogen correlated with the outcome and severity of multiple organ dysfunction in sepsis and disseminated intravascular coagulation (DIC) [1]. Hence, it is widely accepted that PAI-1 can be a useful biomarker for evaluating the severity of sepsis. Furthermore, the over-suppression of fibrinolysis caused mainly by PAI-1 constitutes an important target for therapy in patients with sepsis and DIC [2]. In the former issue of *Journal of Intensive Care*, Hoshino et al. [3] reported the clinical significance of measuring total PAI-1 antigen in sepsis in their retrospective observational study. Here, we introduce the accumulated knowledge with respect to this issue.

## Main body

PAI-1, a 48-kDa serine proteinase inhibitor (SERPIN), produced by various cells such as vascular endothelial cells, platelets, smooth muscle cells, fibroblasts, adipose tissue, and monocytes/macrophages [4], is the main physiological plasminogen activator inhibitor. Like other SERPINs, PAI-1 inhibits its target proteinases, tissue-type plasminogen activator, and urokinase-type plasminogen activator by the formation of a 1:1 stoichiometric reversible complex [5]. PAI-1 can occur in various molecular forms in blood, including active PAI-1 (mainly PAI-1 complexed with vitronectin), inactive or latent PAI-1 (mainly present in lysed platelets and dominant in serum), and PAI-1 complexed with its target proteases (urokinase-type plasminogen activator and tissue-type plasminogen activator). There are several commercialized kits available to detect PAI-1 antigen, and thus, appropriate selection of the kit depending on the purposes is important [6]. Currently, we also can assess the PAI-1 activity, and this will be more suitable to detect the fibrinolytic activity rather than the total produced volume or distribution of PAI-1.

Since the production of PAI-1 is regulated by pro- and anti-inflammatory cytokines such as interleukin-1β, interleukin 6, and transforming growth factor-β, its relation to the inflammatory responses has been strongly assumed [7]. In pathogenesis of sepsis, PAI-1 plays a role in several biological processes dependent on the inhibition of plasminogen activators and plasmin activity [5]. As a consequence, overproduction of PAI-1 may contribute to organ dysfunction in patients suffering from severe infection and DIC.

The fibrinolytic system is much simpler compared to the complexity of the coagulation system. Basically, plasmin is the only activator of fibrinolysis, and the balance between tissue plasminogen activator and PAI-1 mainly regulates plasmin activity. Thrombin-activatable fibrinolysis inhibitor (TAFI) and neutrophil elastase also have the capability to modulate fibrinolysis, but the pathophysiological roles of these factors have not been fully elucidated. Dynamic changes in fibrinolytic status are crucially involved in the pathogenesis of DIC and multiple organ dysfunction in sepsis [8]. Recent evidences indicate that physical entrapment of microbes by fibrin at the site of infection may limit their capacity to disseminate into the systemic circulation. Under these circumstances, impairment of fibrinolysis contributes to the protective role in host defense [9].

In healthy human volunteers, endotoxin infusion induces a rapid change in the coagulation system. Inflammatory cytokines such as tumor necrosis factor and interleukin-6 rose within 120 min with a concurrent rise in the plasminogen activators, indicating endothelial activation. Within 150 min, these changes are counteracted by an even greater and sustained rise in PAI-1 leading to clot longevity [10]. These observations suggest that both activated endothelial cells and platelets cooperate to induce a thrombotic state by producing PAI-1. Finally, the reduction in activated protein C because of reduced availability of thrombomodulin may also play a role in decreased fibrinolysis. Less activated protein C is available to inhibit PAI-1, thus augmenting clot stability.

The clinical significance of measuring PAI-1 in sepsis has been actively studied since 1990s, and it has been repeatedly reported that the elevated PAI-1 levels was associated with an unfavorable outcome in sepsis [11, 12]. Previously, Koyama et al. [13] measured various coagulation/fibrinolysis markers in sepsis patients and reported that thrombin-antithrombin complex (TAT), PAI-1, and protein C discriminated well between patients with and without overt DIC (area under the receiver operating characteristic curve [AUROC], 0.77, 0.87 and 0.85, respectively) and, using the three together, significantly improved the AUROC up to 0.95. Amongst the significant diagnostic markers for overt DIC, TAT and PAI-1 were the best predictors of 28-day mortality (AUROC, 0.77 and 0.81, respectively). Following similar reports, Hoshino et al. [3] reported that the elevated levels of PAI-1 was associated with the poor outcome and the AUROC for 28-day mortality was 0.72, and the optimal cutoff level was 83 ng/mL. What were unique in their observations were firstly, only PAI-1 but no other coagulation markers such as TAT, plasmin-plasmin inhibitor complex, protein C, thrombomodulin, and soluble fibrin were associated with mortality. Secondly, the optimal cutoff was 83 ng/mL [reference range < 50 ng/mL], and that was extremely lower than the previous reports. The detail was not discussed, but these results were a little bit surprising to us. Aforementioned molecular markers and anticoagulants have been repeatedly reported as useful severity markers [14, 15]. One possible reason for the discrepancy is the timing of sampling. Since the coagulation/fibrinolytic change is a dynamic process, the sampling should not be just “ICU admission” but should be specified like the timing from the onset. To make the report more informative, we also would like to suggest to add newer techniques like thromboelastography. Rotational thromboelastography (TEG) and thromboelastometry (ROTEM) are point-of-care tests, which evaluate whole-clot formation and dissociation. It was reported that decreased fibrinolytic activity, as reflected by the lysis index, was found to discriminate the severe cases [16].

In contrast to the regulation of PAI-1, less is known about the plasminogen activation system, its maintenance should be precisely regulated, but the detail still remains to be clarified. Microorganisms including bacteria, fungi, and parasites have been proven to interact in a specific manner with the components of fibrinolysis regulators, i.e., pathogenic microorganisms are capable to destabilize the function of proteases, activators, or inhibitors of fibrinolysis to disseminate in the host and evade from the immune response [17].

Regarding the fibrinolytic system, some other components are known to relate to its function. For example, TAFI plays an important role in the inhibition of fibrinolysis [18]. PAI-2 also inhibits the fibrinolysis and is reported to increase in non-survivors of sepsis [19]. Based on the above knowledge, not only the measurement of fibrinolysis inhibitors but the comprehensive evaluation including activators of fibrinolysis would be important for the accurate understanding of pathophysiology of sepsis.

## Conclusion

It has been clarified that changes of coagulation/fibrinolytic status are deeply related to the development of septic organ dysfunction. Since the status changes dynamically and varies significantly depending on the patients’ condition, comprehensive evaluation is necessary to understand this delicate and complex situation. Based on the accumulated knowledge, we do not recommend the one-point PAI-1 antigen analysis but recommend the sequential analysis of the multi-factors for the accurate assessment of coagulation/fibrinolytic systems. This careful approach may avoid misleading the future studies in this area.

## Abbreviations

AUROC: Area under the receiver operating characteristic curve
DIC: Disseminated intravascular coagulation
PAI-1: Plasminogen activator inhibitor type 1
SERPIN: Serine proteinase inhibitor
TAFI: Thrombin-activatable fibrinolysis inhibitor
TAT: Thrombin-antithrombin complex
